# Suicidality, Economic Shocks, and Egalitarian Gender Norms

**DOI:** 10.1093/esr/jcv084

**Published:** 2016-02-01

**Authors:** Aaron Reeves, David Stuckler

**Affiliations:** Department of Sociology, University of Oxford, Oxford, OX1 3UQ, UK

## Abstract

Durkheim conceived of suicide as a product of social integration and regulation. Although the sociology of suicide has focused on the role of disintegration, to our knowledge, the interaction between integration and regulation has yet to be empirically evaluated. In this article we test whether more egalitarian gender norms, an important form of macro-regulation, protects men and women against suicidality during economic shocks. Using cross-national data covering 20 European Union countries from the years 1991 to 2011, including the recent economic crises in Europe, we first assessed the relation between unemployment and suicide. Then we evaluated potential effect modification using three measures of gender equality, the gender ratio in labour force participation, the gender pay gap, and women’s representation in parliament using multiple measures. We found no evidence of a significant, direct link between greater gender equality and suicide rates in either men or women. However, a greater degree of gender equality helped protect against suicidality associated with economic shocks. At relatively high levels of gender equality in Europe, such as those seen in Sweden and Austria, the relationship between rising unemployment rates and suicide in men disappeared altogether. Our findings suggest that more egalitarian forms of gender regulation may help buffer the suicidal consequences of economic shocks, especially in men.

## Introduction

The sociology of suicide, especially the tradition of scholarship influenced by Durkheim, has tended to focus on the roles of integration and disintegration in suicide risk (Abrutyn and Mueller, 2014). Yet the existing literature has been relatively silent on the importance of regulation, in what Wray and colleagues recently describe in the *Annual Review of Sociology* as the ‘neglected theme of regulation’ (2011: 514). This dearth of evidence is perhaps surprising given that Durkheim conceived of population-level suicides as a product of both integration and regulation.

**Figure 1:**
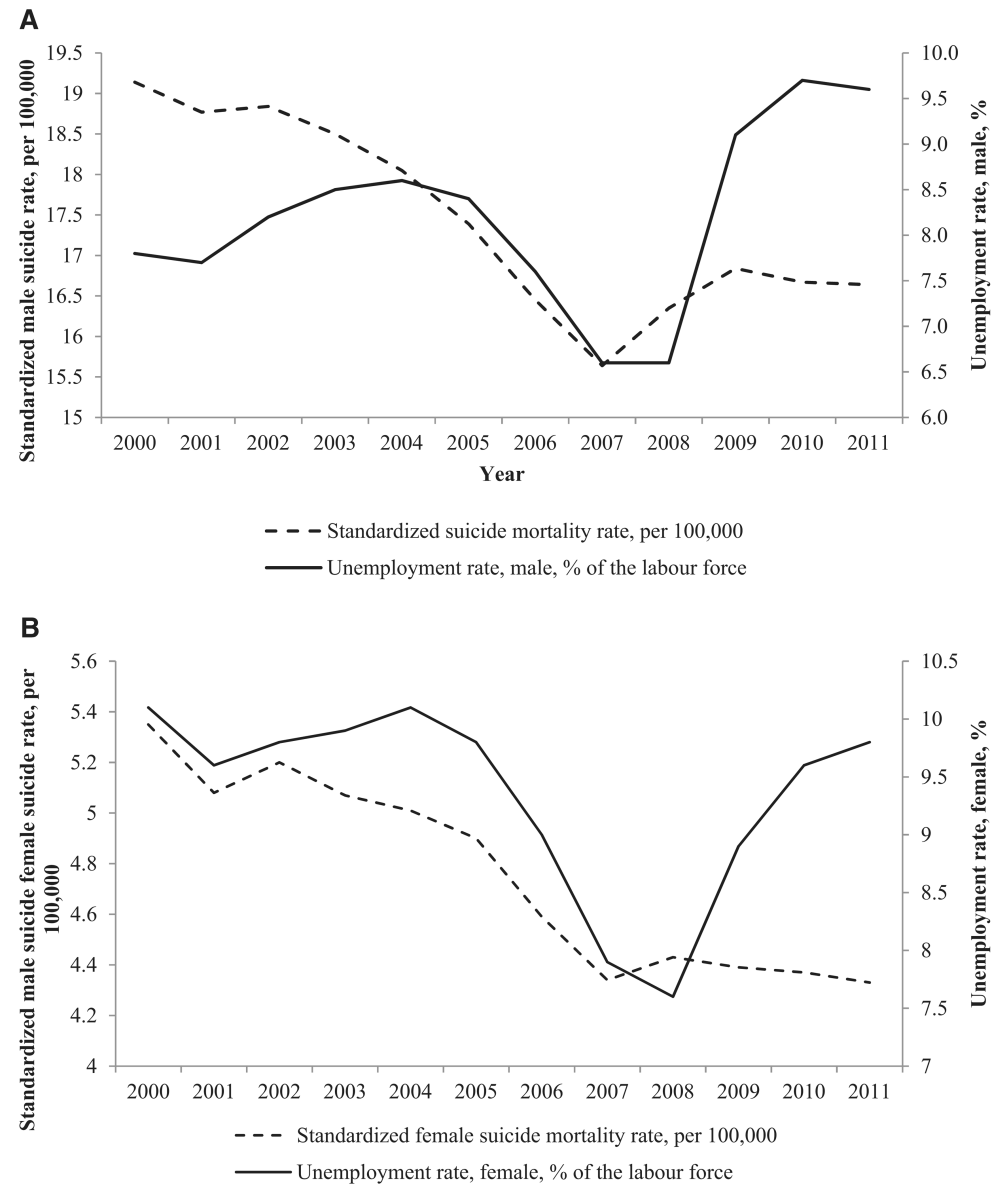
Trends in unemployment rates and the age-standardized suicide rates per 100,000 (A) men and (B) women, 24 EU countries, 2000–2011. *Note:* Luxembourg, Malta, and Cyprus are excluded. *Source*: WHO Health for All European Mortality database 2013 edition; EuroStat 2013 edition.

Quite possibly the most extensively researched area in the sociology of suicide is on economic crisis. As Durkheim wrote, ‘whenever serious readjustments take place in the social order, whether or not due to a sudden growth or to an unexpected catastrophe, men are more inclined to self-destruction’ (1970: 207). The Great Recessions that beset Europe in 2007 has been the most significant period of economic upheaval since the Great Depression. The recessions led to mass unemployment and, with it, a large jump in suicide rates (Reeves *et al.*, 2014) (Figure 1).

Yet, if these stark rises in suicides during the Great Recession were simply a classic Durkheimian story of *anomie*, it would be tragic, yet unsurprising. On closer inspection, however, Europe’s recent rise in what has been popularly termed ‘economic suicides’ exhibits two curiosities, both which point to the possibility that gender regulation is playing an important modifying role. First, these additional economic suicides were mostly concentrated in men. Long recognized as a gendered phenomenon (Wray *et al.*, 2011), in virtually all countries where data are available, suicide risks are commonly 3- to 4-fold higher in men than women. During the Great Recession, this gap widened: female suicide rates increased only by about 1.4 per cent, whereas male rates jumped by 7.7 per cent (Reeves *et al.*, 2014). Second, the rise in male suicides was not uniform across all countries but was substantially greater in some countries than others and, in some, did not rise at all. For example, male suicide rates increased by 10.1 per cent in the Netherlands, whereas male suicide rates in Germany rose only by 1.3 per cent.

Could more egalitarian gender norms—one form of societal regulation—be protective against suicide? To our knowledge, there is scant empirical work on the role of gender regulation or norms in the scholarship on suicidality. Traditional gendered roles in society, Durkheim argued, would render women relatively immune to suicide because they did ‘not participate in collective life in the same way’ as men because they were infrequent labour market participants (Durkheim, 1970: 307). Hence, they were less likely to be exposed to the modernizing forces, which could lead to economic dislocation. In societies where gender norms are less egalitarian, there may be greater normative pressure on men to fulfil breadwinner roles and so increase the status loss associated with unemployment (Stack, 2000). Such a view has, however, been criticized for drawing on outdated assumptions concerning gender relations and lacks empirical evidence (Pampel, 1998).

Here, we test whether more egalitarian gender norms, measured through economic and political indicators of the gap between men and women, regulates suicidality. Our analysis builds on Durkheim’s seminal work as it continues to shape both contemporary sociological thinking on suicide (Abrutyn and Mueller, 2014; Hoffman and Bearman, 2015) and the cross-national methods used in such studies (Durkheim, 1970). By taking advantage of marked cross-national variations that occurred during the Great Recessions of 2007–2010 in Europe, we demonstrate that men living in societies that hold more egalitarian gender regulation have lower risks of suicide during economic shocks.

### Economic Disintegration, Deregulation, and Suicide

Two central concepts in Durkheim’s theory of suicide—disintegration and deregulation—were poorly defined in his original work and, partly as a result in subsequent scholarship, at times conflated (Johnson, 1965, Wray *et al.*, 2011). Thus, before turning to our analysis, we review the post-Durkheimian literature on suicide to help clarify their meaning (Thorlindsson and Bjarnason, 1998).

Durkheim defined integration as ‘the extent of social relations binding a person or a group to others’ (Bearman, 1991: 503). It refers to a sense of social belonging or inclusion; it is a way of conceptualizing the place that an individual has in the social world. This place can be embedded within a community, which involves rich networks of association, which provide support, or it can involve isolation (Pescosolido and Georgianna, 1989). These integrative networks can be familial, religious, political, or economic and positively correlate with health.

How a person or group is integrated exposes them to Durkheim’s second concept—regulation. To distinguish from integration, regulation refers to the ‘demands placed on the individual that come with membership in a group’ (Bearman, 1991: 503). It is the ‘clarity of norms and sanctions governing those ties’ that integrate (Abrutyn and Mueller, 2014: 329). These norms ‘regulate’ people because they foster behaviours that align with them and inhibit those which deviate.

When people experience the loss of or change in their sense of place within a community—becoming disintegrated—they may feel disoriented and hopeless about the future, leading to anomie and associated suicidal thoughts (Wray *et al.*, 2011). As Durkheim explained, disintegration breaks the ‘temporal bond of union’ and ruptures the ‘society’ in which an individual has forged some degree of reciprocity and commonality (Durkheim, 1970: 113). This disintegration may be accompanied by deregulation—that is, a lack of clarity regarding the norms of the group—which leads to a state that Durkheim referred to as anomie, where a person is ‘abruptly cast’ from their ‘accustomed status’ through circumstances beyond their immediate control (1970: 248).

One major form of both disintegration and deregulation arises from economic shocks, which involve a transition from economic security to economic insecurity. One primary form of economic shock is rising job loss. Although Durkheim explicitly connected job loss with deregulation, job loss is frequently invoked as a form of Durkheimian disintegration, in both public health (Chang *et al.*, 2013) and sociology (Norstrom, 1995). For example, Abrutyn and Mueller use job loss to illustrate disintegration because it weakens ‘the quantity and quality of social ties’ and deregulation because it inevitably obscures the ‘norms and sanctions governing those ties’ (Abrutyn and Mueller, 2014: 329). In Europe, job loss does lead to a radical reformulation of social networks thereby disrupting previous forms of integration (Gallie, 1999). Put simply, employment is a central way that people become integrated into the collective order, and so rising unemployment rates involve disintegration because it cuts people off from a particular social world, a set of ties.

It may be this dual shock of disintegration and deregulation which makes job loss so harmful to mental health. To take just two examples: one record linkage study from the United Kingdom found that between 1981 and 1992, unemployed persons, irrespective of gender, were 2.6 times more likely to commit suicide than the employed (Lewis and Sloggett, 1998). Longitudinal data from Denmark from 1981 to 1997 observed that unemployment increased the risk of suicide for both men and women (Qin *et al.*, 2003).

Yet, the precise mechanisms by which economic shocks increase suicide risk, as well as how to prevent it, are not well understood. Durkheim (1970) emphasized the loss of status through disruption to the collective life, stating that ‘if … industrial or financial crises increase suicides, this is not because they cause poverty, since crises of prosperity have the same result; it is because they are crises, that is, disturbances of the collective order’ (1970: 206). This disintegration can be particularly harsh for men because employment is a critical part of masculine identities in some cultures, and hence, it is also one explanation for why suicides are higher in men than women (Cleary, 2012).

This leads to the classic Durkheimian *anomie* hypothesis:

#### Hypothesis 1: Economic shocks will increase suicide risks, particularly in working-age men

##### Gender equality, regulation, and suicide

A missing aspect of previous scholarship is how regulation may mitigate suicidality. This approach builds on the insight of van Tubergen *et al.* (2005) that ‘the impact of the level of integration on suicide risk is conditional … on the norms’ of the group, such as gender norms. Durkheim too highlighted that gender norms were central features of regulation, but he stopped short of specifying particular mechanisms in the social production of suicidality.

It has been speculated that more egalitarian gender regulation can increase suicidality, albeit in different ways for men and women. In societies where gender norms are economically egalitarian, women are more likely to participate in the labour market. This would increase their exposure to economics shocks through unemployment (leading to disintegration and deregulation), directly increasing their suicide risk. Previous research indicates that when female labour force participation rises, their suicide rates initially increase (Pampel, 1998).

**Figure 2:**
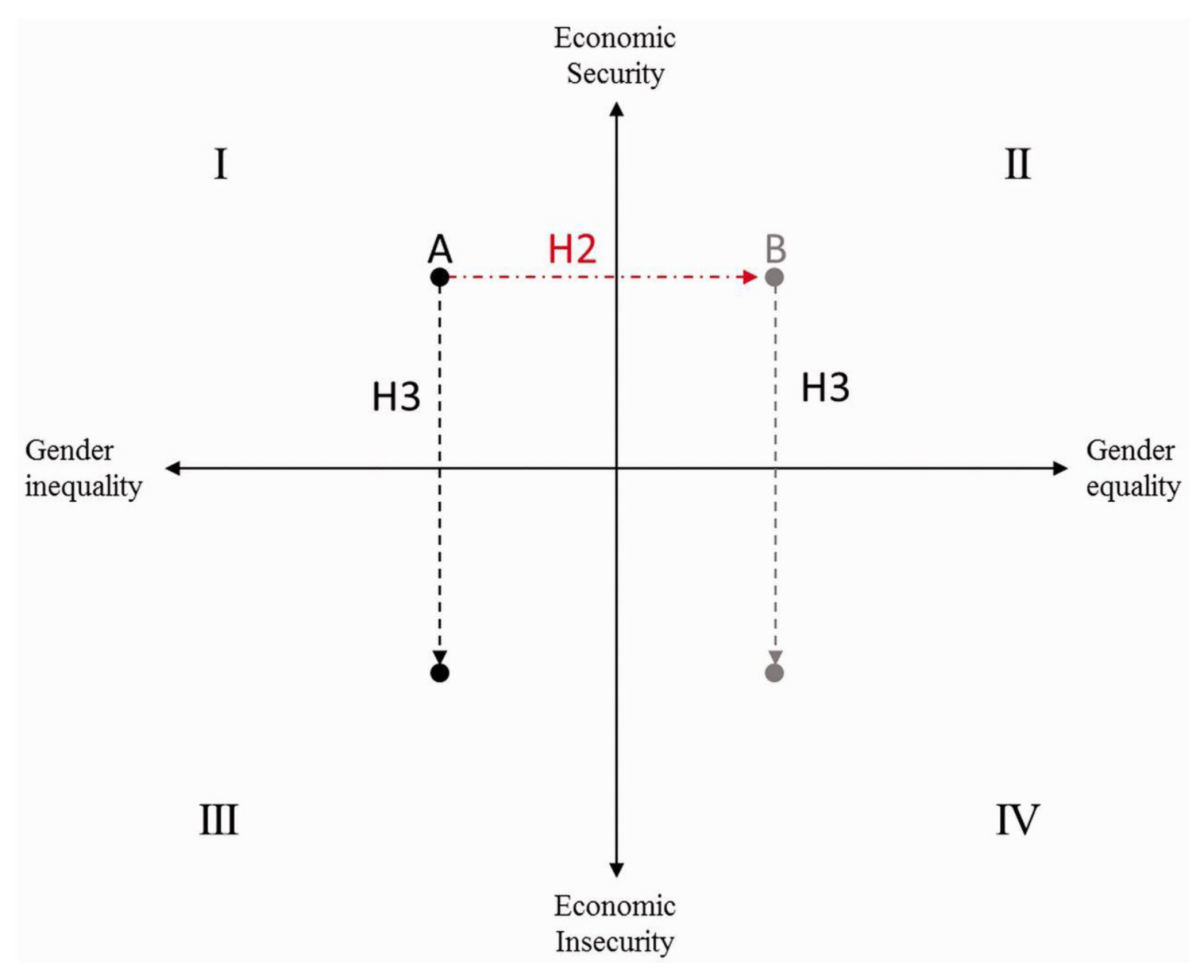
Conceptual model of the association between the economic shocks and gender regulation with suicide. *Note:* The transition from economic security to economic insecurity is what constitutes an economic shock.

To capture this dynamic, in Figure 2 we represent the association between economic shocks and gender regulation. As shown in the figure, on the upper left-hand side (quadrant I), person A lives in a country with less egalitarian gender norms, while person B, on the upper right-hand side (quadrant II), lives in a country with more egalitarian gender norms. We hypothesize that suicide risk will differ in countries with egalitarian gender norms (quadrant II) and countries with less egalitarian norms (quadrant I), irrespective of the level of economic security (see Figure 2 – H2).

#### Hypothesis 2: Countries with egalitarian gender norms, especially in the labour market, will have greater female suicides

Durkheim further stressed that integration and regulation, ‘may simultaneously affect the same individual and impose their combined effects upon [them]’ (1970: 251). In this view, it is important to consider not just one or the other but the product of integration and regulation together. Turning again to Figure 2, if both person A and person B become increasingly likely to lose their job and now face economic insecurity, e.g., experience an economic shock, but at differ levels of gender equality, we hypothesize that:

#### Hypothesis 3: Men who lose jobs in countries with greater gender equality will have lower risks of suicides than those in countries with lesser gender equality

This hypothesis is consistent with previous evidence that while both men and women have better mental health in gender egalitarian societies, men also appear to benefit more than women (Hopcroft and Bradley, 2007, Van de Velde *et al.*, 2013). In the context of suicide specifically, this may be because negative mental health consequences of job loss may be greater for men in societies where gender norms are less egalitarian and where traditional conceptions of masculinity predominate. Evidence from settings as diverse as Ghana (Adinkrah, 2012) and Ireland (Cleary, 2012) suggests that ‘gender norms and the pursuit of masculinity ideals impact suicidal behavior’, especially when men breach norms of economic responsibility (Adinkrah, 2012: 480). These norms of masculinity can constrain the ability of men to express themselves emotionally to seek help (Cleary, 2012). The demands placed on men will also vary, influencing how men respond to economic shocks (Schrijvers *et al.*, 2012). The loss of status for men associated with unemployment and the greater financial burden of being the sole breadwinner will be higher in countries where these traditional notions of masculinity are more common (Hausmann *et al.*, 2006). Web Appendix 1 further summarizes how more egalitarian gender structures may shape the link of disintegration and suicide.

## Method and Data

### Cross-National Suicide Data

We collected age-standardized suicide rates covering the years 1991–2011 from the World Health Organization’s (WHO) Human Mortality Database, 2013 edition. Twenty-four European Union (EU) countries were included in the study. Malta, Luxembourg, and Cyprus were excluded owing to missing data and small populations.

Earlier studies of suicide, such as those of Douglas (1967), drew attention to how social and cultural meanings around suicide (e.g., religious pressure on local governments to conceal suicides in official data) may bias reporting of suicide in official statistics. Particularly relevant to our study, Douglas argued that misreporting may create spurious associations with so-called social determinants of suicide. Critics of recent work in public health examining the influence of the Great Recession on suicide have frequently used this biased-reporting argument to raise doubts about these findings (Reeves *et al.*, 2014). Although caution is required when comparing suicide rates across countries, a recent review of classification errors found that this concern is often overstated (Wray *et al.*, 2011).

Nonetheless, to help overcome potential classification errors, we based suicide rates on the International Classification of Diseases, 10th revision (ICD-10) classification codes X60-84 and ICD-9 classification E950-959, which include intentional injuries via poisoning, hanging, drowning, firearms and explosives, jumping from a height, or other methods. Any residual bias created by these cultural factors should be non-differential with respect to changes within countries over the short periods of economic disintegration our study investigates (Reeves *et al.*, 2014). As a robustness check, we also incorporated deaths from undetermined injuries and causes, which can include suicides that were not ruled as such through narrative verdicts.

To measure economic shocks among specific age groups, sociologists have frequently used annual percentage changes in unemployment rates (capturing ‘job loss’ and short-term dislocation), which are one of the strongest statistical correlates of suicide rates in cross-national research (Norstrom, 1995, Stuckler *et al.*, 2009). Unemployment also captures the effects of economic recessions felt by households and individuals better than gross domestic product (GDP) and other production-based measures (Reeves *et al.*, 2015). We collected unemployment data from the Organisation for Economic Co-operation and Development’s (OECD) Labour Force Statistics database (2013 edition), based on national labour market surveys and measuring the unemployed persons as people who are out of work and seeking a job, as a percentage of the workforce. Age-specific (25–64 years) unemployment rates were only available for 20 countries. The final set of countries included in the models are Austria, Belgium, Czech Republic, Denmark, Estonia, Finland, France, Germany, Greece, Hungary, Ireland, Italy, the Netherlands, Poland, Portugal, Slovak Republic, Slovenia, Spain, Sweden, and the United Kingdom. As a robustness check, we also analysed changes in unemployment rates from 24 countries using data from EuroStat (ages 25–75 years), which has a less precise match with the age brackets of the WHO’s suicide data (ages 25–64 years). These models also include Bulgaria, Latvia, Lithuania, and Romania.

### Cross-National Gender Norm Data

Researchers use attitudinal and non-attitudinal measures of gender norms. The former typically includes subjective views of the primacy of the breadwinner role, the presence of gendered spheres, and balance of parental roles within the household (Davis and Greenstein, 2009). These attitudinal measures have been critiqued for failing to disentangle whether such self-reports capture personal beliefs or attitudes toward prescribed roles (Davis and Greenstein, 2009). Some countries may have highly egalitarian gender norms but still exhibit barriers to gender equality. Hence, we evaluate non-attitudinal indicates that measure observable outcomes of gender equality, such as increased female labour force participation, greater female political representation, and a smaller gender pay gap, which tend to better facilitate cross-national comparisons.

In following with the tradition of using non-attitudinal measures, we operationalize egalitarian gender norms using the mean value of a measure gender equality derived from World Economic Forum (Hausmann *et al.*, 2006). This measure covers both political and economic spheres, areas that are highly correlated with more egalitarian gender norms more generally (Hausmann *et al.*, 2006). The World Economic Forum scales have been extensively used in comparative studies of educational attainment, political enfranchisement, fertility levels, leadership, corruption, and social movements (e.g., Fryer and Levitt, 2010). From this widely cited measure of gender equality, we combine indicators of the gender ratio in labour force participation, the gender pay gap, and representation in parliament, scaled from 0—perfect inequality to 10—perfect equality. This measure does not capture more or less regulation but rather qualitatively different forms of regulation.

We expand on previous research by moving beyond female labour force participation as a measure of gender equity (Pampel, 1998) and examine a range of economic and political dimensions of gender regulation (Davis and Greenstein, 2009). Economic equality includes increased labour force participation, equal pay between the sexes, and changing expectations around childcare. Political equality is expressed through female suffrage, participation in local, regional, and national political governing bodies (such as parliaments), and public policies reflect female concerns and issues. Yet, one limitation of this measure is that it does not necessarily capture the split between the private and public sphere of social life as characteristics of the gender organization of reproduction and production. Despite this limitation, we argue that combining these measures enables us to capture the degree to which gendered regulatory norms are egalitarian (Davis and Greenstein, 2009).

Aside from this indicator, other measures of gender equality also exist. As a sensitivity test, we use another measure of gender equality, developed specifically for European countries by the European Institute for Gender Equality (EIGE), to ensure our results are consistent across similar indicators. The EIGE measure incorporates a broader set of indicators across these same political, economic, and normative dimensions of gender egalitarianism. The EIGE covers the same set of European countries available to us using the gender equality index from the World Economic Forum (Bonfils *et al.*, 2013). We also conduct a sensitivity test using a purely attitudinal measure of gender norms, derived from questions in the World Values Survey, to test whether the prevalence of egalitarian attitudes moderates the unemployment–suicide association. This measure has fewer countries but does provide a useful test of the robustness of our findings to alternative measures of egalitarian gender norms. Table 1 provides further descriptive statistics for all variables used in the study.

### Statistical Modelling

In the first step of the analysis, we quantify the influence of economic shocks on suicide by estimating the effects of changes in male and female unemployment rates on annual changes in age-standardized male and female suicide rate, respectively, for persons aged 25–64 years, 65+ years, and all ages.

(1) $$\begin{array}{cc} \Delta {\mathrm{Suicide}}_{\mathrm{sijt}}= & \beta_{0}+\beta_{1}\Delta {\mathrm{UE}}_{t}+{\mathrm{year}}_{t}+{\mathrm{country}}_{i}\\ & \times {\mathrm{year}}_{t}+\varepsilon_{\mathrm{sijt}} \end{array}$$

Here *s* is gender, *i* is country, *j* is age category (25–64 years, 65+ years, and total), and *t* is the year. *Suicide* is the annual percentage change in the suicide rate (Δ denotes yearly change), *UE* is our measure of economic shocks, measured as a percentage-point change in the sex-specific unemployment rate. We also adjust for time-dummies and country-specific time trends.

Then, we test our second hypothesis by modelling the influence of gender equality (i.e., regulation) on between-country differences in suicide rates across nations.

(2) $${\mathrm{Suicide}}_{\mathrm{sit}}=\beta_{0}+\beta_{1}{\mathrm{Gender}}_{i}+{\mathrm{GDP}}_{\mathrm{sit}}+{\mathrm{year}}_{t}+\varepsilon_{\mathrm{sit}}$$

As above, *s* is gender, *i* is country, and *t* is the year. *Gender* is the direct effect of gender equality on the change in the suicide rate. The measure of gender equality has been standardized so that a 1-unit increase in gender equality is equal to 1 standard deviation (SD). Because suicide rates between countries and gender equality is correlated with economic wealth we also include *GDP* as a control variable, adjusted for purchasing power and inflation. We also adjust for time dummies.

Finally, we examine whether specific types of regulation modify the influence of disintegration on suicide by investigating whether gender equality modified the association between unemployment and suicide, yielding the following equation:

(3) $$\begin{array}{cc} \Delta {\mathrm{Suicide}}_{\mathrm{sijt}}= & \beta_{0}+\beta_{1}\Delta {\mathrm{UE}}_{\mathrm{sijt}}+\beta_{2}{\mathrm{Gender}}_{i}\\ & +\beta_{3}\Delta {\mathrm{UE}}_{\mathrm{sijt}}\times {\mathrm{Gender}}_{i}+{\mathrm{year}}_{t}\\ & +{\mathrm{country}}_{i}\times {\mathrm{year}}_{t}+\varepsilon_{\mathrm{sijt}} \end{array}$$

Here *s* is gender, *i* is country, *j* is age category (25–64 years, 65+ years, and total), and *t* is the year. *Suicide* is the annual percentage change in the suicide rate (Δ denotes yearly change), *UE* is our measure of economic shocks, measured as a percentage-point change in sex-specific unemployment rate. *Gender* is the direct effect of gender equality on the change in the suicide rate. β_3_ is the coefficient of interest; the modifying effect of gender equality on the unemployment–suicide association. This captures the extent to which gender equality increases resilience to economic shocks. We also adjust for time dummies and country-specific time trends.

Coefficients were transformed as semi-elasticities to facilitate interpretation, which here express a percentage change in the dependent variable associated with a percentage point change in an independent variable. First-differencing the data, although a conservative approach, reduces potential bias arising from omitted variables, which are more likely to correlate with trends in the dependent variable than with annualized changes, and other forms of endogeneity, because differenced independent variables are independent of the differenced error term. Additionally, as noted above, by focusing on the change within countries we remove any between-country differences in how suicides are reported, thereby reducing the extent to which our results may be biased by such cultural differences in suicide reporting. As a robustness check, we used dynamic fixed-effects regression to model both the long-term trend and the impact of a short-term economic shock. All models were estimated using STATA version 13.

## Results

First we assess the well-known link between unemployment and suicide (Hypothesis 1) and then we examine whether female suicides rates are higher in countries with high levels of gender equality (Hypothesis 2). Finally, we test whether men who lose jobs in countries with greater gender equality will have lower risks of suicides than those in countries with lesser gender equality (Hypothesis 3).

### Effects of Unemployment and Gender Equality on Male and Female Suicide

**Figure 3:**
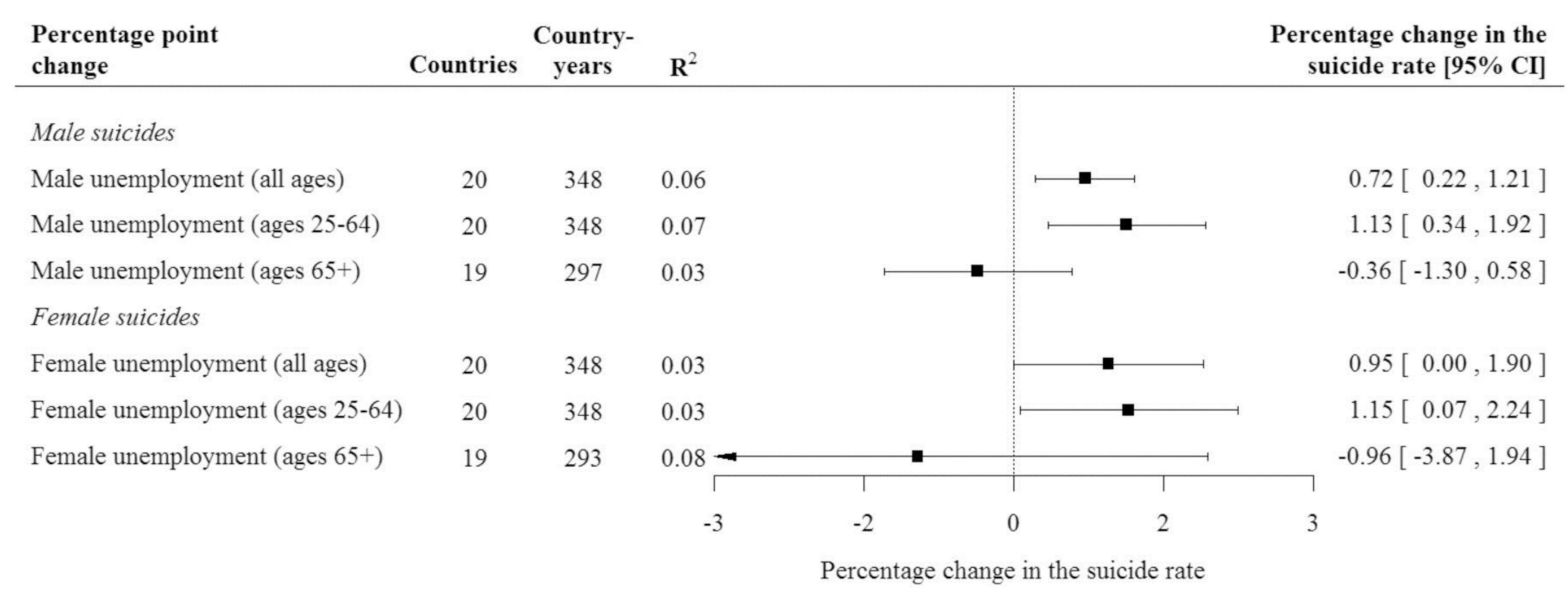
Unemployment and suicide, by gender, 20 EU countries, 1991–2011. *Note:* Luxembourg, Malta, and Cyprus are excluded. *Source*: WHO Health for All European Mortality database 2013 edition; OECD 2013 edition. Male and female unemployment both measured as a 1 per cent point increase. All models estimate the association of the change in the suicide rate with the corresponding age band. For example, the change in the male suicide rate for ages 25–64 years is regressed on the change in the male unemployment rate for the same ages. All models correct for year- and country-specific time trends. Confidence intervals are based on standard errors corrected for repeated observations.

The forest plot in Figure 3 shows the results from the cross-national fixed-effect models for unemployment and suicide. Consistent with previous work, we find that a 1 percentage-point rise in male unemployment is linked with a 0.72 per cent rise in the male suicide rate (95 per cent confidence interval [CI]: 0.22 to 1.21 per cent). This association was most pronounced in working-age men, in whom a 1 percentage-point rise in unemployment among men aged 25–64 years is associated with a 1.13 per cent rise in the male suicide rate among this same age group (95 per cent CI: 0.34 to 1.92 per cent). As anticipated, there is no association between rising male unemployment on suicide risk in persons aged ≥65 years, who are more likely to be retired and outside the labour market. As shown in Figure 3, similar patterns and effect sizes were observed in females.

To test the specificity of our findings (‘falsification test’), we examine whether changes in unemployment predict changes in mortality from Alzheimer’s disease. Without a clear link between unemployment and Alzheimer’s, we would expect to find no association between these variables. We find, as anticipated, that increased unemployment among men aged 25–64 years had no association with Alzheimer’s mortality (*P* = 0.18) nor did female unemployment among the same group (*P* = 0.30).

Having demonstrated that economic shocks, as a form of disintegration and deregulation, increases suicide risk in both men and women, we next tested our second hypothesis of whether countries with more egalitarian gender norms had higher rates of female suicides. Table 2 shows the results of a pooled ordinary least squares model examining variation in suicide rates *between* countries with differing levels of gender equality. We use a between-country comparison because most European countries, i.e., those nations included in this analysis, were fairly stable over this period. For example, between 2006 and 2011, the United Kingdom saw a small improvement (0.098), but Sweden saw a small decline (−0.089). Contrary to hypothesis 2, we found no effect of egalitarian gender norms on male or female suicide (Table 2).

To examine whether our results are consistent across similar measures of gender equality, we re-estimate these models using a distinct but related measure of egalitarian gender norms. This alternative measure of gender equity comes from the EIGE and includes many of the same indicators that constitute the World Economic Forum’s measure. As a result of the European focus, the range of indicators used to construct the EIGE measure is far broader and therefore provides a useful test case (Bonfils *et al.*, 2013). Importantly, the EIGE covers the same sample of countries included in our main models using the Global Gender Index. Again comparing the gender equality–suicides association between countries, we observe that there was no relationship between egalitarian gender norms and male or female suicide (see Web Appendix 2).

### Testing Effect Modification of Gender Equality

**Figure 4:**
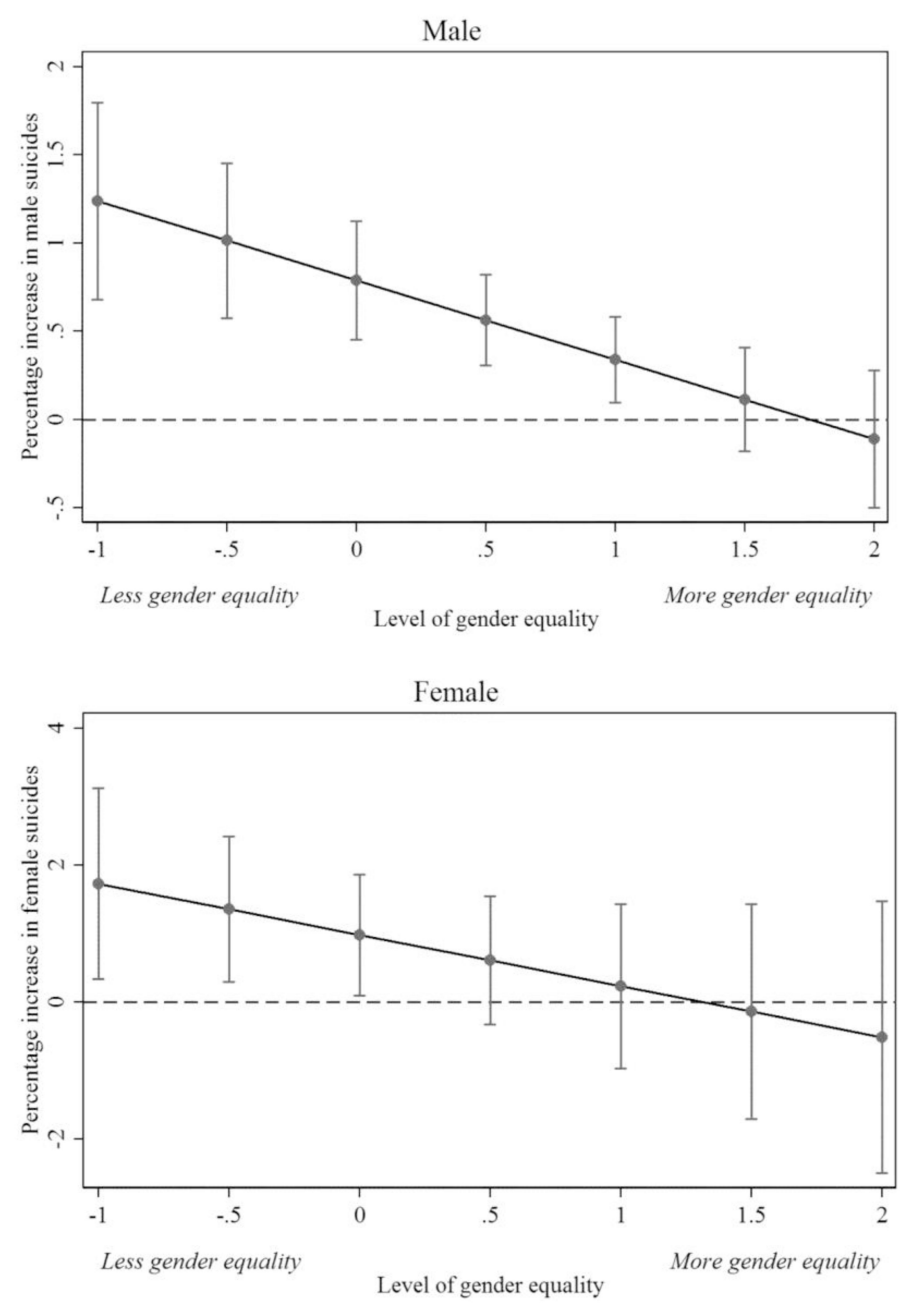
The association between unemployment and suicide declines as countries become more gender equal. *Note:* Figures drawn from estimates in Table 4. To interpret the level of gender equality (measured as z-scores), the following are representative countries: Bulgaria ~ −0.5, Hungary ~ −1, Austria ~ 0, the Netherlands ~ 0.5, Denmark ~ 1, Finland ~ 2.

In the final step, we tested the neo-Durkheimian hypothesis that gender regulation moderated suicide risk associated with economic shocks, such as rising unemployment rates. Table 3 shows that each 1 SD increase in the degree of gender equality reduced the male suicide rate by 0.45 per cent (95 per cent CI: −0.18 to −0.72 per cent). This would reduce the estimated effect of a 1 percentage-point rise in male unemployment on suicide in half (Figure 4).

To put the magnitude of these differences in gender equality in perspective, this shift would be approximately similar to Estonia achieving levels of gender equality seen in Sweden. Conversely, a 1 SD increase in gender equality had no association with the female suicide rate (0.75 per cent: 95 per cent CI: 0.21 to −1.70 per cent) (Figure 4).

When disaggregated by age, we found that the moderating effect of gender equality was the strongest among working men (−0.54 per cent, 95 per cent CI: −0.029 to −1.05 per cent). No effect was seen in women, irrespective of the age group (Table 4). In sum, our evidence was consistent with hypothesis 3, that more egalitarian gender norms reduced suicide risk, particularly for men, linked to economic disintegration.

To test the specificity of our findings, we examine whether gender regulation moderated the risk of Alzheimer’s-related mortality associated with economic shocks. Given that Alzheimer’s mortality rates are unaffected by unemployment, as documented earlier, we would anticipate that egalitarian gender norms would not moderate this relationship. As expected, gender equality does not moderate the unemployment–suicide association for men (*P* = 0.20) or women (*P* = 0.30).

Finally, we again use an alternative measure of gender equity from the EIGE to test whether our findings are consistent across different indicators of egalitarian gender norms. We find that our results are slightly attenuated when using the EIGE indicator but the coefficients are not qualitatively different between models for both men (test of effect homogeneity χ^2^ (1) = 1.32, *P* = 0.25) and women (test of effect homogeneity χ^2^ (1) < 0.01, *P* = 0.98) (Web Appendix 3).

### Robustness Checks

We performed a series of checks on our sample and model specification. Our main analysis is restricted to the 20 countries for which we have OECD employment data. To enhance the sample size, we re-estimated our models using EuroStat data on unemployment, adding Latvia, Lithuania, and Romania to the sample (Web Appendix 4). None of our results were substantively altered by including these additional countries. GDP, adjusted for inflation and purchasing power, might confound the association between gender equality, unemployment, and suicide. To test this hypothesis, we include a measure of the level of GDP in our models and found that our results again do not qualitatively change (Web Appendix 5).

Additionally we used dynamic fixed-effects regression to model both the long-term trend and the impact of a short-term shock on that trend (i.e., a rise in unemployment) while estimating the impact of the gender equality effect modifier for both men and women. Consistent with our previous models we found that higher gender equality reduces the association between male unemployment and male suicide. Likewise, higher gender equality does not alter the association between female unemployment and female suicide (see Web Appendices 6 and 7).

We also estimated the effect of these modifiers controlling for suicide prevention programmes and found that the coefficients did not substantively change (see Web Appendix 8) (Matsubayashi and Ueda, 2011). Because our measure of suicide may underreport the actual number of deaths in some countries, we examined whether our results were consistent with a combined measure of (i) suicide and (ii) deaths of ‘unknown’ causes. When using this combined measure, the sample size is reduced but the direction and magnitude of the coefficients are similar across models (see Web Appendix 9). Position in the life course—such as age and, relatedly, whether someone is married or has children—may alter the association between economic shocks and suicide (Girard, 1993, Maimon and Kuhl, 2008, Umberson *et al.*, 2010). To explore this possibility, we test the association between unemployment for those aged 15–24 years and the suicide rate among those aged 15–29 years. Our results indicate that the effect of gender equality on youth male suicide is attenuated compared with the main effect, but it remains significant at the α = 0.05 level (see Web Appendix 10). Marital integration may moderate the association between gender equality and the unemployment–suicide association (Gibbs, 2000). Again, we find that moderating effect of gender equality on the unemployment–suicide rates remains relatively stable after controlling for the marriage rate and the divorce rate (Web Appendix 11).

Gender segregation in the labour market is an important dimension in these debates, but, unfortunately, data in this area are limited. To explore this area more fully, we have now adjusted our models for parental leave (institutional support for female labour market participation) and the proportion of women in part-time work (which is often also low-paid and measures the feminization of poverty). Again, we find that our results remain unchanged after adjusting for these variables (Web Appendix 12).

Finally, we test whether egalitarian gender norms as measured in the World Values Survey moderates the unemployment–suicide association in a similar way to our measure of gender egalitarianism. We find similar results to our main models even though we have fewer countries (and therefore fewer observations) in this sample (Web Appendix 13).

## Discussion and Conclusion

Taken together, these results stress the importance of examining the ‘neglected theme of regulation’ in the sociology of suicide. Building on the work on previous suicide researchers that have documented the impact of economic shocks, such as rising unemployment rates, on suicide, this study endeavours to refocus attention on regulation in the sociological study of suicide.

Specifically, we have argued that it is crucial to examine how both disintegration and regulation jointly influence the suicide risk of a population. This approach moves beyond existing work that finds rising unemployment rates increases suicide risk to demonstrate that this association is not inevitable and critically modified by other regulatory forces—specifically more egalitarian gender norms. These findings help explain why the unemployment–suicide association varies between countries (Reeves *et al.*, 2014). In short, during periods of disintegration and deregulation (such as rising unemployment), regulatory gender norms can either exacerbate or constrain suicidality. Three conclusions can be drawn from these results.

First, when unemployment rises so does the risk of suicide, particularly among working-age men and women. What remains unclear from this and other studies is the reason why unemployment increases suicidality. One explanation is that unemployment increases financial strain leading to suicide. Durkheim, however, did not believe this material deprivation explanation, which he suggested ‘may even be considered a protection’ (Durkheim, 1970: 206). Instead, he argued that the association between economic shocks and suicidality was a status effect: i.e., job loss creates a gap between current and previous status positions leading to anomie. While more research is needed, our results are consistent with this status explanation because the moderating impact of gender egalitarianism is consistent with a status, rather than material mechanism.

Second, we find that egalitarian gender norms have no direct association with suicide risk for women. Female labour force participation may create some incongruity between female norms and behaviour that could, in turn, increase female suicide risk. But this relationship appears to be temporary because female labour participation often precedes changes in institutional structures and social norms that ‘catch up’ to create a ‘a more supportive environment for the new roles of women’ (Pampel, 1998: 746). In short, when changes to institutions and normative expectations occur, they foster a ‘more supportive environment’, removing the incongruity between norms and behaviour, and then suicide rates decline to their previously low levels (Pampel, 1998). Recent European data are consistent with this view, where, in some countries, women with high economic power—such as those who are employed—can experience poorer health than women with low economic power (Van de Velde *et al.*, 2010). In such countries, employed women are more likely to (i) receive unequal pay and/or (ii) face the dual burden of wage and domestic labour. Consistent with these previous studies, our results suggest that egalitarian gender norms do not increase male or female suicide rates when there is greater economic equality and ‘a more supportive’ institutional and normative environment.

Third, egalitarian gender norms mitigate the influence of unemployment on suicide in men through reducing the prevalence of the male breadwinner culture, which, in turn, reduces exposure to suicide risk factors linked with that specific culture of masculinity (see Web Appendix 1). Cross-national, psychological evidence using personality tests indicates that differences between men and women on five major personality traits increase in countries with more egalitarian gender norms, suggesting men are more able to express themselves without the constraints of hegemonic masculinity (Schmitt, 2015). These findings may help explain patterns of behaviour that increase suicide risk across countries. For example, where egalitarian gender norms regulate social interaction, we find more help-seeking during periods of psychological stress, thereby reducing suicide risk. Egalitarian gender norms may also reduce the likelihood that men will consume more alcohol following job loss. Symptoms of depression are also less common in both sexes when regulatory gender norms are more equitable, although men seem to benefit more than women (Hopcroft and Bradley, 2007, Van de Velde *et al.*, 2013). One further implication of Van de Velde *et al.*’s (2013) study is that the gains for men of gender equality may be inequitably distributed among men. Finally, in cultures where traditional ‘breadwinner’ norms are less dominant, the financial pressures associated with job loss and indebtedness are less focussed on men (Cleary, 2012). Importantly, this protective effect of gender equality does not come at the expense of women, where we find no detrimental effects of egalitarian gender norms on female suicide.

Egalitarian gender relations almost completely remove the impact of unemployment on male suicide by altering the regulatory norms that shape responses to economic shocks (Figure 4). Our results confirm Durkheim’s key insight that regulation and integration both ‘simultaneously affect the same individual and impose their combined effects upon [them]’ (1970: 251). This framework could also illuminate other areas where macro-social regulatory norms and integration may affect cross-national variation in suicidality, e.g., variation in the alcohol–suicide association and the divorce–suicide association. In addition to this contribution, this article also provides a modelling framework through which such hypotheses can be tested.

As with any statistical analysis, this study has several limitations. First, owing to limited data, we used a constant measure of gender equality over time to capture different regulatory norms. This reduces our ability to examine the influence of changes in gender equity on suicide over the past 20 years. Because our measure of gender equity is based on data from the 2000s, after many of the newer EU member states had made great strides toward gender equity, these data offer conservative estimates of the differences between countries. Second, while data on unemployment is widely available, other measures, such as underemployment and precarious employment (which has been increasing rapidly in some countries), may be as important for suicide risk.

Third, drawing individual-level inferences from ecologic data is potentially at risk of committing the ecological fallacy. For example, we cannot identify that those who experience suicide are also those losing their job. Among those who lost their job, we also cannot observe whether disintegration, deregulation, or both was the mechanism. Like van Tubergen and colleagues (van Tubergen *et al.*, 2005), we view Durkheim’s work within a micro–macro approach, suggesting that gender norms shape the relationship between employment status and suicide at the individual level. Thus, to understand population level risks, we need to study population-level risk factors.

Fourth, the measure of gender equality contains measurement error because it does not perfectly capture the degree to which gender norms are egalitarian. Yet, assuming the error is non-differential with respect to EU countries, any error will undermine our ability to observe an association. Fifth, our findings may not generalize universally, although they are consistent with previous global studies of the unemployment–suicide association (Chang *et al.*, 2013). Because our sample is drawn from Europe, we believe the observed relationships likely hold in other advanced industrialized nations.

These findings have important implications. First, according to our results, this rise in male suicides during the Great Recession has been concentrated in countries with less egalitarian gender norms. These findings indicate that economic uncertainty does not necessarily lead to a widening of the gender gap in suicide rates during recessions, especially where regulatory gender norms are egalitarian. If the male suicide rate had risen in line with the (lower) female suicide rate, then there would have been 2,380 fewer male suicides in Europe (Reeves *et al.*, 2014). Second, there is little evidence that egalitarian gender norms increase male or female suicide rates, especially in societies where men and women are equal across various domains of social life. Policies and programmes that increase gender equality, such as reducing the gender pay gap, may not only be valuable in their own right, but a critical strategy for protecting men and women against the adverse consequences of economic shocks.

## Supplementary Material

Web Appendix

## Figures and Tables

**Table 1:** Detailed description of key variables

Variables	Mean (SD)	Min	Max	Country-years	Countries	Coding	Source
Male suicide rate, per 100,000	−0.31 (11.65)	−48.09	108.58	516	25	Annual percentage change	WHO-HRA European Mortality Database 2013 edition
Male suicide rate, ages 25–64 years, per 100,000	−0.17 (13.16)	−54.36	83.45	508	25	Annual percentage change	WHO-HRA European Mortality Database 2013 edition
Male suicide rate, ages 65+ years, per 100,000	0.75 (27.12)	−79.31	322.29	505	25	Annual percentage change	WHO-HRA European Mortality Database 2013 edition
Male unemployment rate (%)	0.08 (1.35)	−6.30	11.05	726	21	Annual percentage point change	OECD Labour database 2013 edition
Male unemployment rate (%), ages 25–64 years	0.09 (1.20)	−5.79	10.32	726	21	Annual percentage point change	OECD Labour database 2013 edition
Male unemployment rate (%), ages 65+ years	0.00 (1.37)	−12.05	10.77	642	19	Annual percentage point change	OECD Labour database 2013 edition
Female suicide rate, per 100,000	−1.48 (12.36)	−42.96	68.32	619	25	Annual percentage change	WHO-HRA European Mortality Database 2013 edition
Female suicide rate, ages 25–64 years, per 100,000	−1.19 (15.11)	−51.62	112.96	591	25	Annual percentage change	WHO-HRA European Mortality Database 2013 edition
Female suicide rate, ages 65+ years, per 100,000	0.39 (34.56)	−73.82	626.27	612	24	Annual percentage change	WHO-HRA European Mortality Database 2013 edition
Female unemployment rate (%)	0.06 (1.36)	−5.31	6.73	524	21	Annual percentage point change	OECD Labour database 2013 edition
Female unemployment rate (%), ages 25–64 years	0.11 (1.25)	−5.04	7.76	524	21	Annual percentage point change	OECD Labour database 2013 edition
Female unemployment rate (%), ages 65+ years	−0.05 (3.46)	−30.43	26.67	455	19	Annual percentage point change	OECD Labour database 2013 edition
Gender equality^a^	2.44 (1.47)	0.57	6.16	644	27	Re-scaled to 0–10	World Economic Forum

*Note*:

a Gender equality index: this measure of gender equality has been produced by the World Economic Forum and consists of measures with respect to four key indicators (each measure indicator is measured on a scale of 0 to 1): (1) Economic participation and opportunity, including the rates of participation, gendered pay gaps, and the number of women in key professional positions, (2) Political empowerment is measured through the ratio of women to men in ministerial or parliamentary positions, (3) Educational attainment is measured through the ratios of men to women in primary-, secondary-, and tertiary-level education, and (4) Health and survival measures sex ratio at birth and the gap between male and female life expectancy. This collection of measures is intended to be independent of the level of wealth in a particular country.

**Table 2:** Association between gender equality and suicide, all ages, 1991–2011

Covariate	Male suicide (per 100,000)	Female suicide (per 100,000)
1 SD increase in gender equality	0.026 (−4.72 to 4.77)	0.68 (−0.39 to 1.74)
USD 100 increase in level of GDP per capita	−0.14 (−0.61 to 0.33)	0.015 (−0.066 to 0.097)
Country-years	258	258
Countries	23	23

*Note:* Confidence intervals are based on robust standard errors clustered by country.

*Source*: WHO Health for All European Mortality database 2013 edition; OECD 2013 edition; World Economic Forum, 2013 edition. All models control for year dummies. One SD increase in gender equality is the equivalent of Estonia becoming like the Netherlands.

* *P*<0.05;

** *P*<0.01.

**Table 3:** Effect of gender equality on the unemployment–suicide association, all ages, 1991–2011

Modifier	Effect size (95% CI)	*P*-value
Men	Percentage change in the male suicide rate	
1 SD increase in gender equality	−0.45%** (−0.72 to −0.18)	0.003
Country-years	348	
Countries	20	

**Modifier**	**Effect size (95% CI)**	***P*-value**
Women	Percentage change in the female suicide rate	
1 SD increase in gender equality	−0.75% (−1.70 to 0.21)	0.12
Country-years	348	
Countries	20	

*Note:* Confidence intervals are based on robust standard errors clustered by country.

*Source*: WHO Health for All European Mortality database 2013 edition; OECD 2013 edition; World Economic Forum, 2013 edition. All models control for year- and country-specific time trends. Effect sizes are based on modelling the interaction between changes in unemployment and the level of gender equality: β1 × Unemployment + β2 Unemployment × Equality + β3 × Equality. One SD increase in gender equality is the equivalent of Estonia becoming like the Netherlands.

* *P*<0.05;

** *P*<0.01.

**Table 4:** Effect of gender equality on the unemployment–suicide association, ages 25–64 years, 1991–2011

Modifier	Effect size (95% CI)	*P*-value
Men	Percentage change in the male suicide rate	
1 SD increase in gender equality	−0.54%* (−1.05 to −0.029)	0.039
Country-years	348	
Countries	20	

**Modifier**	**Effect size (95% CI)**	***P*-value**
Women	Percentage change in the female suicide rate	
1 SD increase in gender equality	−0.28% (−1.51 to 0.95)	0.64
Country-years	348	
Countries	20	

*Note:* Confidence intervals are based on robust standard errors clustered by country.

*Source*: WHO Health for All European Mortality database 2013 edition; OECD 2013 edition; World Economic Forum, 2013 edition. All models control for year- and country-specific time trends. Effect sizes are based on modelling the interaction between changes in unemployment and the level of gender equality: β1 × Unemployment + β2 Unemployment × Equality + β3 × Equality. One SD increase in gender equality is the equivalent of Estonia becoming like the Netherlands.

* *P*<0.05;

** *P*<0.01.
