# Supplementary material for: Suicidality, Economic Shocks, and Egalitarian Gender Norms
Source: Eur Sociol Rev. Author manuscript; Available in PMC 2016 Feb 11. (PMC4750292; doi:10.1093/esr/jcv084)
Supplement: Web Appendix [file NIHMS66452-supplement-Web_Appendix.pdf]

Web Appendix

Web Appendix 1: Mechanisms linking less egalitarian gender norms and suicide risk

Web Appendix 2: Association between egalitarian gender norms and suicide using the European Institute for Gender Equality measure, all ages, 1991-2011

Web Appendix 3: Effect modifiers of the unemployment-suicide association using European Institute for Gender Equality, all ages, 1991-2011

Web Appendix 4: Effect gender equality on the unemployment-suicide association, all ages, using EuroStat data, 1991-2011

Web Appendix 5: Effect gender equality on the unemployment-suicide association, all ages, controlling for GDP, 1991-2011

Web Appendix 6: Dynamic fixed-effects panel model of the effect modifier of gender equality and male unemployment on the male suicide rate, 20 EU countries, 1991-2011.

Web Appendix 7: Dynamic fixed-effects panel model of the effect modifier of gender equality and female unemployment on the female suicide rate, 20 EU countries, 1991-2011.

Web Appendix 8: Effect gender equality on the unemployment-suicide association controlling for suicide prevention programmes, all ages, 1991-2011

Web Appendix 9: Effect modifiers of the unemployment-suicide association (including unknown deaths), all ages, 1991-2011

Web Appendix 10: Effect of gender equality on the unemployment (16-24)-suicide (15-29) association, 1991-2011

Web Appendix 11: Effect of gender equality on the unemployment-suicide association adjusting for marriage and divorce rates, all ages, 1991-2011

Web Appendix 12: Effect of gender equality on the unemployment-suicide association adjusting for parental leave and the proportion of women in part-time work, all ages, 1991-2011

Web Appendix 13: Effect of egalitarian gender norms on the unemployment-suicide association, 1991-2011

Web Appendix 1: Mechanisms linking less egalitarian gender norms and suicide risk

Consequences of less egalitarian gender norms	Short-term Outcome	Long-term outcome	Citations
Traditional masculinity more common			
Men less likely to recognise and more likely to hide symptoms of depression	Less help-seeking	Increased suicide risk	De Leo D, Cerin E, Spathonis K, Burgis S. Lifetime risk of suicide ideation and attempts in an Australian community: prevalence, suicidal process, and help-seeking behaviour. J Affect Disord 2005; 86(2-3): 215-24.
Men more likely to be violent toward other men	More perpetrators and victims of violence	Increased suicide risk among victims and perpetrators	Archer J. Cross-cultural differences in physical aggression between partners: A social-role analysis. Pers Soc Psychol Rev 2006; 10(2): 133-53.<br>Whaley RB, Messner SF. Gender Equality and Gendered Homicides. Homicide Studies 2002; 6(3): 188-210.
More loss of status for men following unemployment	More disintegration for men	Increased suicide risk for men	Chung A. Gender difference in suicide, household production and unemployment. Appl Econ 2009; 41(19): 2495-504.
Larger gender wage gap			
More intimate partner violence toward women	More women victims of violence	Increased suicide risk among women	Devries KM, Mak JY, Bacchus LJ, et al. Intimate partner violence and incident depressive symptoms and suicide attempts: a systematic review of longitudinal studies. PLoS Med 2013; 10(5):

			e1001439.
More women in poverty	Increased financial strain	Increased suicide risk among women	Van de Velde S, Bracke P, Levecque K. Gender differences in depression in 23 European countries. Cross-national variation in the gender gap in depression. Soc Sci Med 2010; 71(2): 305-13.
Less female labour force participation			
Increased economic burden of unemployment for men	Increased financial strain	Increased suicide risk among men	Reeves A, McKee M, Gunnell D, et al. Economic shocks, resilience, and male suicides in the Great Recession: cross-national analysis of 20 EU countries. European Journal of Public Health 2014.

Web Appendix 2: Association between egalitarian gender norms and suicide using the European Institute for Gender Equality measure, all ages, 1991-2011

Covariate	Male suicide (per 100,000)	Female suicide (per 100,000)
1 standard deviation increase in gender equality	-2.36 (-8.45 to 3.73)	0.69 (-0.31 to 1.68)
USD$100 increase in level of GDP per capita	-0.014 (-0.38 to 0.41)	-0.001 (-0.066 to 0.064)
Country-years	269	269
Countries	24	24

Notes: Confidence intervals are based on robust standard errors clustered by country. Source: WHO Health for All European Mortality database 2013 edition; OECD 2013 edition; European Institute for Gender Equality, 2013 edition. All models control for year dummies. One standard deviation increase in gender equality is the equivalent of Estonia becoming like the Netherlands.

* p-value<0.05; ** p-value<0.01

Web Appendix 3: Effect modifiers of the unemployment-suicide association using European Institute for Gender Equality, all ages, 1991-2011

**Men**	Percentage change in the male suicide rate	
Modifier	Effect size (95% CI)	p-value
1 standard deviation increase in gender equality	-0.33% (-0.73 to 0.06)	0.09
Country-years	348	
Countries	20	
**Women**	Percentage change in the female suicide rate	
Modifier	Effect size (95% CI)	p-value
1 standard deviation increase in gender equality	-0.74% (-1.75 to 0.26)	0.14
Country-years	348	
Countries	20	

Notes: Confidence intervals are based on robust standard errors clustered by country. Source: WHO Health for All European Mortality database 2013 edition; OECD 2013 edition; European Institute for Gender Equality, 2013 edition. All models control for year and country-specific time trends. Effect sizes are based on modeling the interaction between changes in unemployment and the level of gender equality. $\beta 1$xUnemployment + $\beta 2$UnemploymentxEquality + $\beta 3$*Equality.

* p-value<0.05; ** p-value<0.01

Web Appendix 4: Effect gender equality on the unemployment-suicide association, all ages, using EuroStat data, 1991-2011

**Men**	Percentage change in the male suicide rate	
Modifier	Effect size (95% CI)	p-value
1 standard deviation increase in gender equality	-0.42%** (-0.65 to -0.18)	0.001
Country-years	343	
Countries	23	
**Women**	Percentage change in the female suicide rate	
Modifier	Effect size (95% CI)	p-value
1 standard deviation increase in gender equality	-0.18% (-1.34 to 0.97)	0.75
Country-years	343	
Countries	23	

Notes: Confidence intervals are based on robust standard errors clustered by country. Source: WHO Health for All European Mortality database 2013 edition; EuroStat 2013 edition; World Economic Forum, 2013 edition. All models control for year and country-specific time trends. Effect sizes are based on modeling the interaction between changes in unemployment and the level of gender equality. β1xUnemployment + β2UnemploymentxEquality + β3*Equality. One standard deviation increase in gender equality is the equivalent of Estonia becoming like the Netherlands.
* p-value<0.05; ** p-value<0.01

Web Appendix 5: Effect gender equality on the unemployment-suicide association, all ages, controlling for GDP, 1991-2011

**Men**	Percentage change in the male suicide rate	
Modifier	Effect size (95% CI)	p-value
1 standard deviation increase in gender equality	-0.49%** (-0.77 to -0.21)	0.002
Country-years	318	
Countries	20	
**Women**	Percentage change in the female suicide rate	
Modifier	Effect size (95% CI)	p-value
1 standard deviation increase in gender equality	-0.46% (-1.53 to 0.60)	0.37
Country-years	318	
Countries	20	

Notes: Confidence intervals are based on robust standard errors clustered by country. Source: WHO Health for All European Mortality database 2013 edition; OECD 2013 edition; World Economic Forum, 2013 edition. All models control for year and country-specific time trends. Effect sizes are based on modeling the interaction between changes in unemployment and the level of gender equality. β1xUnemployment + β2UnemploymentxEquality + β3*Equality. One standard deviation increase in gender equality is the equivalent of Estonia becoming like the Netherlands.

* p-value<0.05; ** p-value<0.01

Web Appendix 6: Dynamic fixed-effects panel model of the effect modifier of gender equality and male unemployment on the male suicide rate, 20 EU countries, 1991-2011.

Dynamic fixed-effects model	Percentage change in the male suicide rate<br>(1)
**Long-run trends**	
Year˚	-1.69%**<br>(-1.60 to -1.78)
**Short-run effects**	
Error-correction speed of adjustment parameterˆ	-0.97%**<br>(-0.25 to -1.70)
Percentage point increase in male unemployment	0.81%**<br>(0.58 to 1.03)
1 standard deviation increase in gender equality	-0.27%*<br>(-0.04 to -0.50)
Country-years	359
Countries	20

Notes: Source: WHO Health for All European Mortality database 2013 edition; OECD 2013 edition; World Economic Forum, 2013 edition. * p-value<0.05; ** p-value<0.01 Standard errors in parentheses are adjusted for repeated observations. Controls include: country-specific trends and the level of gender equality index.
˚Country-specific time trends (not reported here) deviate from the EU wide time trend.
ˆ The error correction speed of adjustment coefficient is the estimate of how fast, in percentage terms, the suicide rate returns to the long-term trend after a short-term shock. Our estimates suggest that there is a 2.15% reduction in the male suicide rate each year after the initial rise in unemployment.

Web Appendix 7: Dynamic fixed-effects panel model of the effect modifier of gender equality and female unemployment on the female suicide rate, 20 EU countries, 1991-2011.

Dynamic fixed-effects model	Percentage change in the male suicide rate (1)
**Long-run trends**	
Year˚	-13.07%* (-1.90 to -13.07)
**Short-run effects**	
Error-correction speed of adjustment parameterˆ	-0.24%* (-0.0028 to -0.49)
Percentage point increase in female unemployment	0.89%* (0.025 to 1.76)
1 standard deviation increase in gender equality	-0.82% (0.21 to -1.85)
Country-years	359
Countries	20

Notes: Source: WHO Health for All European Mortality database 2013 edition; OECD 2013 edition; World Economic Forum, 2013 edition. Standard errors in parentheses are adjusted for repeated observations. Controls include: country-specific trends and the level of gender equality index.
˚Country-specific time trends (not reported here) deviate from the EU wide time trend.
ˆ The error correction speed of adjustment coefficient is the estimate of how fast, in percentage terms, the suicide rate returns to the long-term trend after a short-term shock. Our estimates suggest that there is a 2.15% reduction in the male suicide rate each year after the initial rise in unemployment.
* p-value<0.05; ** p-value<0.01

Web Appendix 8: Effect gender equality on the unemployment-suicide association controlling for suicide prevention programmes, all ages, 1991-2011

	Percentage change in the male suicide rate	
Modifier	Effect size (95% CI)	p-value
1 standard deviation increase in gender equality	-0.45%**<br>(-0.72 to -0.18)	0.003
Country-years	348	
Countries	20	
	Percentage change in the female suicide rate	
Modifier	Effect size (95% CI)	p-value
1 standard deviation increase in gender equality	-0.44%<br>(-0.93 to 0.046)	0.073
Country-years	348	
Countries	20	

Notes: Confidence intervals are based on robust standard errors clustered by country. Source: WHO Health for All European Mortality database 2013 edition; OECD 2013 edition. Suicide Prevention Programmes, Matsubayashi & Ueda, 2011; World Economic Forum, 2013 edition. All models control for year and country-specific time trends. Effect sizes are based on modeling the interaction between changes in unemployment and the level of gender equality. $\beta 1$xUnemployment + $\beta 2$UnemploymentxEquality + $\beta 3$*Equality. One standard deviation increase in gender equality is the equivalent of Estonia becoming like the Netherlands. Suicide prevention programmes equals 0 if a country initiated no nationwide suicide prevention program between 1980 and 2004.

* p-value<0.05; ** p-value<0.01

Web Appendix 9: Effect modifiers of the unemployment-suicide association (including unknown deaths), all ages, 1991-2011

**Men**	Percentage change in the male suicide rate	
Modifier	Effect size (95% CI)	p-value
1 standard deviation increase in gender equality	-0.48% (-1.53 to 0.58)	0.36
Country-years	240	
Countries	20	
**Women**	Percentage change in the female suicide rate	
Modifier	Effect size (95% CI)	p-value
1 standard deviation increase in gender equality	-0.34% (-1.54 to 0.86)	0.56
Country-years	240	
Countries	20	

Notes: Confidence intervals are based on robust standard errors clustered by country. Source: WHO Health for All European Mortality database 2013 edition; OECD 2013 edition; World Economic Forum, 2013 edition. All models control for year and country-specific time trends. Effect sizes are based on modeling the interaction between changes in unemployment and the level of gender equality. β1xUnemployment + β2UnemploymentxEquality + β3*Equality. One standard deviation increase in gender equality is the equivalent of Estonia becoming like the Netherlands. Dependent variable is a combined measure of suicide deaths and deaths of unknown cause.

* p-value<0.05; ** p-value<0.01

Web Appendix 10: Effect of gender equality on the unemployment (16-24)-suicide (15-29) association, 1991-2011

**Men**	Percentage change in the 15-29 male suicide rate	
Modifier	Effect size (95% CI)	p-value
1 standard deviation increase in gender equality	-0.27%** (-0.52 to -0.01)	0.040
Country-years	335	
Countries	20	
**Women**	Percentage change in the 15-29 female suicide rate	
Modifier	Effect size (95% CI)	p-value
1 standard deviation increase in gender equality	-0.89% (-2.15 to 0.36)	0.15
Country-years	335	
Countries	20	

Notes: Confidence intervals are based on robust standard errors clustered by country. Source: WHO Health for All European Mortality database 2013 edition; OECD 2013 edition; World Economic Forum, 2013 edition. All models control for year and country-specific time trends. Effect sizes are based on modeling the interaction between changes in unemployment and the level of gender equality. β1xUnemployment + β2UnemploymentxEquality + β3*Equality. One standard deviation increase in gender equality is the equivalent of Estonia becoming like the Netherlands.
* p-value<0.05; ** p-value<0.01

Web Appendix 11: Effect of gender equality on the unemployment-suicide association adjusting for marriage and divorce rates, all ages, 1991-2011

**Men**	Percentage change in the male suicide rate	
Modifier	Effect size (95% CI)	p-value
1 standard deviation increase in gender equality	-0.46%**<br>(-0.74 to -0.17)	0.004
Country-years	333	
Countries	20	
**Women**	Percentage change in the female suicide rate	
Modifier	Effect size (95% CI)	p-value
1 standard deviation increase in gender equality	-0.80%<br>(-1.74 to 0.13)	0.09
Country-years	333	
Countries	20	

Notes: Confidence intervals are based on robust standard errors clustered by country. Source: WHO Health for All European Mortality database 2013 edition; OECD 2013 edition; World Economic Forum, 2013 edition. All models control for marriage rates, divorce rates, and year and country-specific time trends. Effect sizes are based on modeling the interaction between changes in unemployment and the level of gender equality. $\beta 1 x Unemployment + \beta 2 Unemployment x Equality + \beta 3 * Equality$. One standard deviation increase in gender equality is the equivalent of Estonia becoming like the Netherlands.

* p-value<0.05; ** p-value<0.01

Web Appendix 12: Effect of gender equality on the unemployment-suicide association adjusting for parental leave and the proportion of women in part-time work, all ages, 1991-2011

**Men**	Percentage change in the male suicide rate	
Modifier	Effect size (95% CI)	p-value
1 standard deviation increase in gender equality	-0.37%** (-0.60 to -0.14)	0.003
Country-years	313	
Countries	18	
**Women**	Percentage change in the female suicide rate	
Modifier	Effect size (95% CI)	p-value
1 standard deviation increase in gender equality	-0.84% (-1.97 to 0.29)	0.13
Country-years	313	
Countries	19	

Notes: Confidence intervals are based on robust standard errors clustered by country. Source: WHO Health for All European Mortality database 2013 edition; OECD 2013 edition; World Economic Forum, 2013 edition. All models control for marriage rates, divorce rates, and year and country-specific time trends. Effect sizes are based on modeling the interaction between changes in unemployment and the level of gender equality. $\beta 1$xUnemployment + $\beta 2$UnemploymentxEquality + $\beta 3$*Equality. One standard deviation increase in gender equality is the equivalent of Estonia becoming like the Netherlands.
* p-value<0.05; ** p-value<0.01

Web Appendix 13: Effect of egalitarian gender norms on the unemployment-suicide association, 1991-2011

We use two questions from the World Values survey pertaining to gender norms: 1) Men should have more right to a job than women (Agree/Neither/Disagree) and 2) Men make better political leaders than women do (Agree/Disagree). We measured the proportion of the population who disagreed with these statements and created a combined score (Cronbach's alpha = 0.81). We selected these two measures because they covered the widest range of European countries for which we had data (13 countries). We then estimated our interaction models to test whether the association is congruent with the models reported in the paper (see table below).

**Men**	Percentage change in the male suicide rate	
Modifier	Effect size (95% CI)	p-value
10 percentage point increase in the proportion of the population who have egalitarian gender attitudes	-0.26%*<br>(-0.44 to -0.07)	0.012
Country-years	242	
Countries	13	
**Women**	Percentage change in the female suicide rate	
Modifier	Effect size (95% CI)	p-value
10 percentage point increase in the proportion of the population who have egalitarian gender attitudes	-0.50%<br>(-1.22 to 0.22)	0.16
Country-years	242	
Countries	13	

Notes: Confidence intervals are based on robust standard errors clustered by country. Source: WHO Health for All European Mortality database 2013 edition; OECD 2013 edition; World Economic Forum, 2013 edition. World Values Survey. All models control for year and country-specific time trends. Effect sizes are based on modeling the interaction between changes in unemployment and the level of gender equality.
$\beta 1$xUnemployment + $\beta 2$UnemploymentxEquality + $\beta 3$*Equality.
* p-value<0.05; ** p-value<0.01
